# Optimizing antifungal therapy: a systematic review of pharmacist interventions, stewardship approaches, and outcomes

**DOI:** 10.3389/fmed.2024.1489109

**Published:** 2024-12-03

**Authors:** Zunaira Akbar, Muhammad Aamir, Zikria Saleem

**Affiliations:** ^1^Department of Pharmacy, The University of Lahore, Lahore, Pakistan; ^2^Riphah Institute of Pharmaceutical Sciences, Riphah International University, Lahore, Pakistan; ^3^Department of Pharmacy Practice, Faculty of Pharmacy , Bahauddin Zakariya University, Multan, Pakistan

**Keywords:** antifungal, stewardship, pharmacist, consumption, clinical, interventions

## Abstract

**Introduction:**

Specific evidence regarding the pharmacist’s role in antifungal stewardship (AFS) is emerging. This review aims to identify pharmacist-driven AFS interventions to optimize antifungal therapy.

**Methods:**

A systematic review was conducted using Preferred Reporting Items for Systematic Reviews and Meta-Analyses (PRISMA) guidelines. Data (2018–2023) were collected through Google Scholar and PubMed. The collected data were presented descriptively due to variations in interventions and outcome metrics. Conclusions were derived through a qualitative synthesis of the identified findings.

**Results:**

A total of 232 articles were retrieved, and after applying inclusion and exclusion criteria, 27 were included in the review. Among the eight studies evaluating the impact of pharmacist interventions on antifungal consumption, 6 studies reported a significant decline in defined daily dose (DDD)/1,000 patient days and days of therapy (DOT)/1,000 patient days, one reported a non-significant decrease, and one reported an increase in the utilization of echinocandins. Educational intervention was the most commonly used stewardship approach. Nineteen studies reported data on various clinical outcomes. Mortality and length of hospital stay remain non-significant, but the occurrence of ADR decreased significantly, and the quality of antifungal use improved significantly.

**Conclusion:**

Pharmacist-led AFS has the potential to enhance the effectiveness of antifungal treatments by improving their overall quality, reduction in consumption, and adverse events. The healthcare system should encourage multidisciplinary collaboration where pharmacists play a central role in decision-making processes regarding antifungal use.

## 1 Introduction

Invasive fungal infections (IFIs) predominantly involving invasive candidiasis and aspergillosis represent a dynamic and growing global public health concern due to increased risk of morbidity and mortality. Patients with solid organ transplant, hematopoietic stem cell transplant, malignancy, critically ill, long-term corticosteroid and antibiotics use are at higher risk of IFIs (1). The incidence of IFIs varies based on the geographical location (2). Globally, over 800 million individuals experience IFIs, with annual mortality rates reaching 1,660,000, comparable to tuberculosis (1,700,000). In Asian countries, the prevalence of IFI is 3–15 times higher than that in the Western nations. Besides the elevated mortality rates ranging from 10 to 49%, IFIs pose significant economic challenges due to extended hospital stays and severe financial consequences (3).

Antimicrobial resistance (AMR) is recognized as a major deterrent to public health systems, impacting not only developing countries but also worldwide (4). Empiric use of antifungals in critically ill patients and immunocompromised patients increases the risk of antifungal resistance (AFR) (5). One of the biggest challenges in clinical practice is the resistance of *Candida* and *Aspergillus* species to azoles, followed by echinocandins (5). The resistance to antifungal agents can have various contributing factors, including host-related or drug-related factors such as inadequate dosing, inaccurate diagnosis, and patient non-compliance. Additionally, microbiological factors, such as genetic mutations in the organism, or a combination of both, may play a role in this multifactorial mechanism (6).

Drug-resistant microorganisms are becoming more prevalent, endangering the capacity to treat common infections and carry out life-saving procedures such as organ transplants and chemotherapy for cancer. Treatment for fungal infections can be challenging, in part because of interactions between drugs that are being prescribed to patients with comorbid infections, such as HIV and cancer. It is especially concerning with multidrug-resistant *Candida auris*, which is one of the fungal pathogens responsible for invasive fungal infections. WHO has developed a list of 19 fungal priority pathogens categorized into three priority groups, namely, critical, high, and medium priority. These fungi are responsible for invasive infections, which are difficult to treat, and there is a high risk of fungal resistance (7).

Establishing an efficient antifungal stewardship program (AFSP) is crucial for managing drug resistance. This program should integrate rapid fungal diagnostics, therapeutic drug monitoring, and clinical intervention teams. The advancement of improved diagnostic tools and strategies is imperative to enable the precise and targeted utilization of antifungals, ensuring the preservation of their effectiveness and reduction in resistance (8). Studies have shown that AFSP significantly improves the quality of antifungal use, antifungal consumption, and clinical outcomes (9). Pharmacist as a member of AFS program plays a pivotal role in promoting rational drug use and optimizing therapy. Several studies have highlighted the positive impact of pharmacist-led interventions on antimicrobial stewardship programs. Still, specific evidence regarding their role in antifungal stewardship is scarce. This systematic review aimed to explore and document the available literature on Pharmacist-led AFS and the impact of these interventions on antifungal consumption and clinical outcomes.

## 2 Methods

### 2.1 Information sources and search strategy

A comprehensive search was carried out to gather data on pharmacist interventions as a member of the antifungal stewardship team on optimizing antifungal use. The literature was searched through Google Scholar using the keywords “Antifungal stewardship and/or antimicrobial stewardship and/or pharmacist interventions and/or consumption and/or quality of antifungal use and/or clinical outcomes” which provided 130 publications; literature search through PubMed that derived 69 publications with mesh terms “Antimicrobial Stewardship” AND “Antifungal Agents” and 33 publications with mesh terms “Antimicrobial Stewardship” AND “Antifungal Agents” AND “Invasive Fungal Infections/Drug Therapy.” All the available data for the period 2018–2023 was searched in October 2023.

### 2.2 Study eligibility criteria

Inclusion criteria: (a) Studies that delineated an AFS program or intervention done by a pharmacist and presented data on antifungal consumption and clinical outcomes within the AFS program; (b) full access to original research articles; and (c) articles in the English language.

Exclusion criteria: (a) Review papers, editorials, abstracts, and duplicate studies were excluded; (b) Studies lacking an intervention; and (c) those not assessing the designated outcome of interest. The outcomes of interest include any stewardship interventions done by pharmacists that impact antifungal consumption and clinical measures (infectious diseases [IDs] consultation, mortality, length of hospital stay, adherence to guidelines, and adverse events).

### 2.3 Study selection and data extraction process

Two researchers independently screened studies by titles and abstracts initially. After screening titles and abstracts, 27 full-text articles that met the inclusion criteria were reviewed by one researcher. Microsoft Excel spreadsheet was used to document information on all of the included variables: study reference, study design, study setting and location, study period, number of patients, type of intervention, study objective, and study outcomes. A second researcher independently reviewed the extracted data. Any disagreement between collected data was resolved through discussion between all authors.

### 2.4 Synthesis of results

The systematic review adhered to the Preferred Reporting Items for Systematic Reviews and Meta-Analyses (PRISMA) checklist (10). The collected data were presented descriptively due to the variations in interventions and outcome metrics. Conclusions were derived through a qualitative synthesis of the identified findings. All primary studies used descriptive statistics for their evaluations, presenting findings as frequencies and percentages without employing an effect measure estimate. Therefore, the study results were organized descriptively into tables, ensuring transparent reporting of the systematic review findings.

### 2.5 Quality assessment

**Figure 1:**
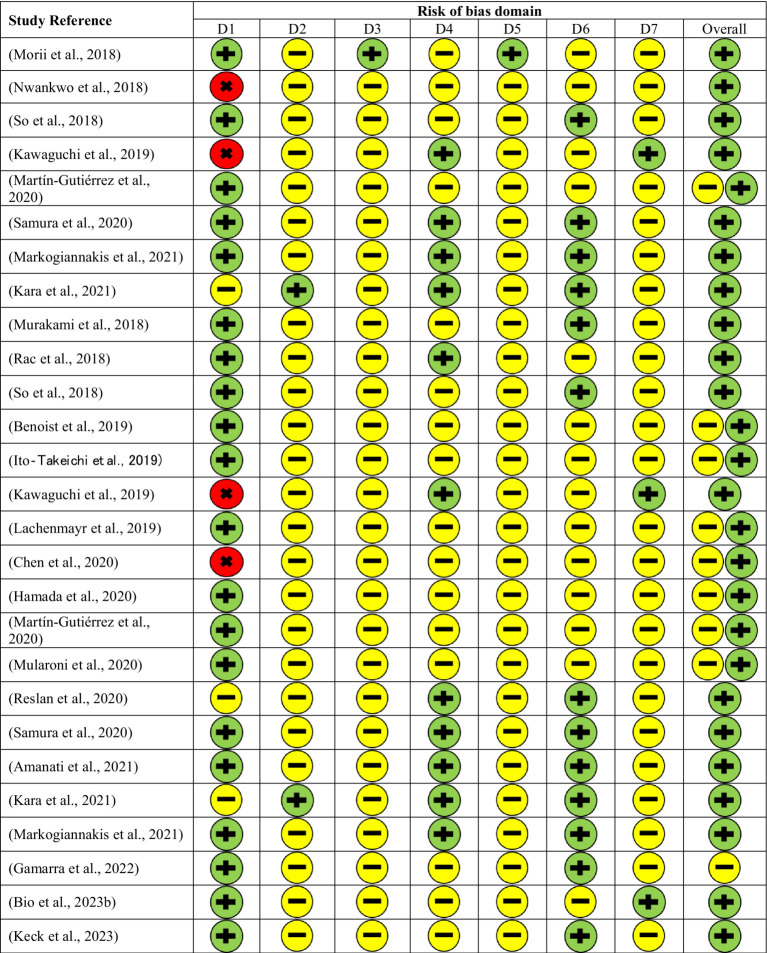
Risk of bias assessment using Risk of Bias In Non-randomized Studies-of Interventions (ROBINS-I) tool. Domains: D1. Bias due to confounding, D2. Bias in the selection of participants in the study, D3. Bias in the classification of interventions, D4. Bias due to deviations from intended interventions, D5. Bias due to missing data, D6. Bias in measurement of outcome, D7. Bias in the selection of the reported result. Judgment: low risk 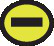, moderate risk 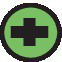, serious risk 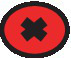.

Three investigators independently assessed the risk of bias in all included studies using the Robin-I tool (11) which evaluates studies across seven domains, including confounding, participant selection, intervention classification, deviation from intended intervention, missing data, measured outcome, and selection of reported results, as shown in Figure 1. The final assessment regarding the risk of bias in all studies was made through a mutual agreement among all authors. Studies were judged as having a low risk of bias if it is comparable to randomized trials, a moderate risk of bias if it provides solid evidence for a non-randomized design, though they cannot be considered equivalent to a well-conducted randomized trial, and a serious risk of bias if it has significant issues that can impact the credibility of results (11). Studies with low or moderate risk were included.

## 3 Results

### 3.1 Search results

**Figure 2:**
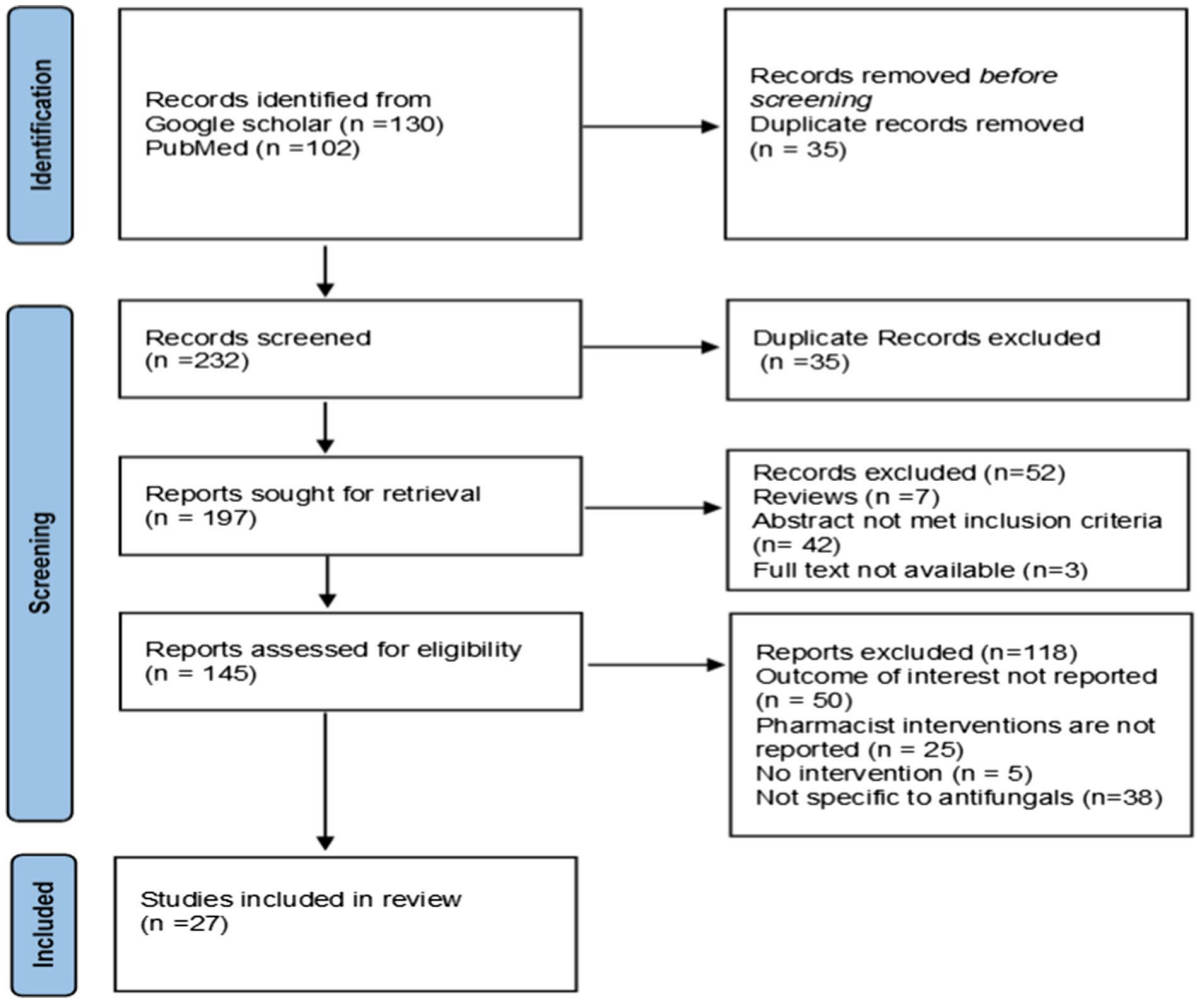
PRISMA 2020 flow diagram.

Two hundred and thirty two articles were identified using keywords and mesh terms through Google Scholar and PubMed. After removing duplicates, abstracts only, review papers, not evaluating pharmacist interventions or the outcome of interest, and not evaluating poststewardship activity impact on outcomes were excluded. After exclusion, 27 articles that met the criteria were included in the systematic review (Figure 2).

### 3.2 Study characteristics

Among 27 studies that are included in the review, eight studies evaluated the impact of pharmacist interventions on antifungal consumption in terms of defined daily dose (DDD) or days of therapy (DOT) and cost reduction. DDD is the assumed average maintenance dose per day of a drug for its primary indication in adults, whereas DOT measures the number of days a patient receives a particular drug, regardless of the dose (12). The remaining 19 studies evaluated the impact of pharmacist interventions on clinical outcomes, including 30-day mortality, length of hospital stay, ID consultations, and occurrence of adverse events, as summarized in Table 1.

**Table 1:** Summary of studies.

Study reference	Study design	Study setting and location	Study population	Study period	Number of patients /prescriptions	Antifungal drug/s	Type of intervention	Study objective/method	Study outcome
*Antifungal consumption (DDD/1,000 patient days; DOT/1,000 patient days; DDD/100 bed days)*									
Morii et al. (33)	Prospective quasi-experimental study-interrupted time series	Showa General Hospital (Tokyo)	Adult in-patients	2006–2016	Not reported	Intravenous (IV) amphotericin B, liposomal amphotericin B, fosfluconazole, miconazole, itraconazole, voriconazole, micafungin, and caspofungin	Educational intervention, prospective audit, and feedback	AF use (DDD/1,000 patient days) and expenditure per fiscal year (FY)	62% decrease in DDD/1,000 patient days and 73% reduction in total expenditure
Nwankwo et al. (34)	Prospective interrupted time series	Tertiary cardiopulmonary care center, Royal Brompton & Harefield NHS Foundation Trust (UK)	Adult in-patients with chronic fungal lung disease	18 months	178 patients	Intravenous antifungals	Recommendations on a diagnostic test, stepping down treatment to the oral drug, conducting TDM, and stopping treatment in patients without a confirmed diagnosis	AF use (DDD/100 bed days) and monthly AF expenditure	Significant reduction in DDD/100 bed days (*p* = 0.017) and 44.8% reduction in expenditure
So et al. (35)	Retrospective observational time series analysis	Tertiary care hospital Princess Margaret Cancer Centre, University Health Network (Canada)	Adult in-patient allogeneic stem cell transplantation in leukemia unit	2005–2013	1,006 patients before ASP, 335 during ASP implementation, internal control (723/264); external control group (1.395/864)	Not reported	Educational intervention. Audit and feedback	Antimicrobial use (DDD per 100 patient days) and cost (Canadian dollar [C$] per patient days)	DDD/100 patient days reduced significantly (*p* < 0.01) and 13.76% reduction in AF expenditure
Kawaguchi et al. (28)	Retrospective, observational	OSAKA City University Hospital, Tertiary care teaching hospital (Japan)	Adults and pediatrics receiving systemic antifungals	2011–2016	978 pre-intervention, 815 in the intervention group	L-AMB, caspofungin, micafungin, fosfluconazole, and voriconazole	Recommendation regarding appropriate selection and modification of AF	AF use (DDD/1,000 patient days), DOT /1,000 patient days.	Reduction in DDD/1000 patient days (NS), DOT/1000 patient days significantly reduced in the interventional group
									(*p* = 0.009), and 13.5% reduction in AF expenditure
Martín-Gutiérrez et al. (36)	Quasi-experimental-interrupted time series	Tertiary care teaching, University Hospital (Spain)	Adult patients ≥18 years with hospital-acquired candidemia	2009–2017	Not reported	Fluconazole, voriconazole, caspofungin, micafungin, anidulafungin and liposomal amphotericin B	Educational intervention including guideline development and periodical clinical sessions	DDD/1,000bed days	38.3% reduction in DDD/1,000 bed days (*p* < 0.001)
Samura et al. (37)	Pre-/post-interventional (cohort)	Yokohama General Hospital (Japan)	Adult Inpatients who developed candidemia	2008–2012	17/20 patients	Intravenous (IV) Fosfluconazole, micafungin, liposomal amphotericin B, and voriconazole, Oral itraconazole, fluconazole, voriconazole	ID pharmacist intervention regarding culture reports and antimicrobial use based on guidelines	Optimal AF drug selection, usage, and expenditure	43.3% reduction in DOT (*p* < 0.001). post-AFS optimal AF usage significantly increased (*p* = 0.025), and AF expenditure significantly decreased (*p* = 0.002).
Markogiannakis et al. (38)	Prospective interrupted time series analysis	A tertiary care teaching hospital, General Hospital of Athens Laiko (Greece)	Adult patients ≥18 years with hematological/oncological malignancy	2015–2017	147 + 138 (285) patients	Azoles, polyenes, and echinocandins antifungals	Educational intervention, prospective audit, and feedback	AF consumption and acquisition cost	23.7% reduction in DDD/1,000 patient (*p* < 0.001) and 26.8% reduction in acquisition cost.
Kara et al. (27)	Prospective quasi-experimental	1,040 bedded Tertiary Care University Hospital (Turkey)	Adult patients ≥18 years	2019–2020	84/101/192 patients	Fluconazole, voriconazole, posaconazole, caspofungin, micafungin, Anidulafungin, Liposomal Amphotericin B and combinations	Educational intervention, audit, and feedback	AF consumption (DOT/1,000 patient days)	59.4 and 60.9% increase in DOT/1.000 patient days for anidulafungin and for caspofungin, respectively, while 33.2 and 41.8% decrease for fluconazole and L-Amphotericin B
*Clinical Outcomes(ID consultations, adherence to guidelines, Length of hospital stay, mortality, Adverse events, others)*									
Murakami et al. (39)	Prospective quasi-experimental	Tertiary Care Saku Central Hospital (Japan)	Patients with candidemia aged >18 years	2006–2012	30 + 46	Micafungin, liposomal amphotericin B, voriconazole, fluconazole	Development of candidemia care bundle based on IDSA guidelines.	30-day all-cause mortality and adherence to guidelines	30-day all-cause mortality 11 (23.9%) vs. 7 (23.3%) NS. Appropriate drug and duration selection (*p* < 0.001), CVC removal following + blood culture (*p* = 0.012), and ophthalmological intervention (*p* < 0.001).
Rac et al. (40)	Prospective Quasi-experimental	Tertiary academic medical center (USA)	Patients ≥18 years having positive culture of candida	2012–2016	50/67	Micafungin and fluconazole	Implementing AF susceptibility testing, culture alerts, removal of central lines	Time to adequate AF therapy, infection-related LOS, compliance to ophthalmology consult, repeat cultures, and ≥ 14 days of adequate therapy	Significant ID consultation (*p* < 0.001), Switch towards oral AF (*p* = 0.015), reduction in time to order antifungal (*p* = 0.017) and receipt of antifungals (*p* = 0.026) post-intervention. No difference in LOS and compliance related to quality indicators in both groups.
So et al. (35)	Retrospective observational time series analysis	Tertiary care hospital Princess Margaret Cancer Centre (PM), University Health Network (Canada)	Adult patients admitted for allogeneic stem cell transplantation in leukemia unit	2005–2013	1,006 patients before ASP, 335 during ASP implementation, internal control (723/264); external control group (1,395/864)	Not reported	Academic detailing (audit and feedback) and ID referrals	LOS, 30-day inpatient mortality	Post-intervention LOS (*p* = 0.29) and in patient mortality (*p* = 0.64) remains NS.
Benoist et al. (16)	Prospective cohort	French university hospital (France)	Adults and pediatric patients isolated with candida	2012-2015	33/37	Micafungin, fluconazole (IV/oral), amphotericin B IV, liposomal amphotericin flucytosine	Recommendation acc. to ESMID guidelines for candidemia management	ID consultations, AF treatment and dose de-escalation, Time to initiate treatment, 30-day and 90-day mortalities	Post-intervention ID consultations increased from 36.4 to 86.5%, with a significant increase in daily blood culture (*p* = 0.04), and dose de-escalation in 52.8% points. Echinocandins usage increased (*p* = 0.03), and 30-day, 90-day mortalities declined from 21.2% vs. 18.9 and 36.4 to 27.0%, respectively (NS).
Ito‐Takeichi et al. (24)	Prospective cohort	Tertiary care, Gifu University Hospital (Japan)	Adult patients with Candidemia infections	2009–2016	35/22	IV AF: Micafungin, fosfluconazole, voriconazole, liposomal Amphotericin B, Caspofungin Oral AF: Fluconazole, Itraconazole, Voriconazole	Prospective audit and feedback along with monitoring βDG measurement	Time of starting therapy, 60-day clinical failure, LOS, and adverse events	Significant decline in clinical failure (*p* < 0.001) 60-day mortality decreased by 42.9% vs. 18.2%, LOS increased from 67 to 85 NS, and significant decline in AE (*p* = 0.004).
Kawaguchi et al. (28)	Retrospective, observational	Tertiary care teaching OSAKA City University Hospital (Japan)	Adult and pediatric patients on systemic antifungals	2011–2016	978/815	L-AMB, caspofungin, micafungin, fosfluconazole, and voriconazole.	Recommendations include selection and modification of AF based on + blood cultures and implementing a candidemia care bundle	30-day mortality, in-hospital mortality, and achievement rates of the candida care bundle	NS decrease in 30-day mortality (*p* = 0.414) In-hospital, mortality reduced significantly (*p* = 0.054) Achievement of the candidemia care bundle was significant (*p* = 0.006).
Lachenmayr et al. (9)	Retrospective observational	University Hospital of Munich (Germany)	Adult patients ≥18 years with hematological/oncological malignancy	2016–2017	103	Liposomal Amphotericin B, voriconazole, posaconazole, fluconazole, caspofungin	Medical training for the physicians and on-ward pharmaceutical counseling	Quality of antifungal through pharmacist interventions and compliance rate of physicians toward interventions	Significant improvement in quality of AF prescriptions (*p* < 0.005) The compliance rate was 66.1%.
Chen et al. (20)	Retrospective observational	2,600 bedded National Taiwan University Hospital (Taiwan)	Adult Patients admitted in hematology department	2014-2016	670/773	Not reported	Interventions in medication orders and therapeutic drug monitoring	Clinical and economic impact of interventions	Pharmacist intervention increased from 0.34 to 1.87% (*p* < 0.00001). LOS reduced from 19.27 to 16.69 Preventable ADE increased from 58 to 230, and 74.7% reduction in cost.
Hamada et al. (23)	Retrospective observational	5 Hospitals (Japan)	Patient ≥18 years	2015–2018	401	voriconazole	Therapeutic drug monitoring	Impact of TDM-based dosing on AE	With dose adjustment, 8/9 patients with hepatotoxicity and 27/28 patients with visual symptoms completed treatment.
Martín-Gutiérrez et al. (36)	Prospective quasi-experimental—interrupted time series	117 beds Tertiary care teaching, University Hospital (Spain)	Adult patients ≥18 years with hospital-acquired candidemia	2009–2017	Not reported	Fluconazole, voriconazole, caspofungin, micafungin, anidulafungin and liposomal amphotericin B	Educational intervention	Incidence of hospital-acquired candida infections and candidemia mortality.	Significant decline in incidence density of hospital-acquired candidemia (*p* = 0.009) and 14-day mortality rate reduced from 36.1 to 19.2% (*p* = 0.3) NS.
Mularoni et al. (41)	Prospective cohort study	Specialized care hospital, ISMETT-IRCCS (Italy)	Adult Patients with invasive fungal infections and solid organ transplant recipient	2009–2018	70	Fluconazole, echinocandins	ID consult for empirical AF and switching from IV to oral fluconazole	Appropriateness of AF use, clinical cure, and costs.	Significant improvement inappropriate antifungal selection (40.5% vs. 78.6%) *p* < 0.0001, Clinical cure was improved 90% vs. 100%. Total expenditure was reduced by 45.8%.
Reslan et al. (32)	Prospective cohort, pre-/postinterventional	Tertiary referral hospital, outpatient cancer center (Sydney)	Out-patients adults >18 years & malignant hematology receiving chemotherapy elderly protocols	2017–2018	40/42	Azoles	Prospective review of prescriptions, suggesting azole TDM, identifying reasons for non-adherence to guidelines	Impact of a weekly pharmacist review on American and New Zealand Consensus Guidelines adherence for antifungal prophylaxis	Appropriate antifungal prophylaxis increased from 31 to 54% (*p* = 0.0001), Appropriate utilization of guidelines increased (*p* = 0.0344). Lack of TDM was the main reason for non-adherence, 48.5% vs. 46%.
Samura et al. (37)	Prospective cohort, pre-/postinterventional	Yokohama General Hospital (Japan)	Adult inpatients who developed candidemia	2008–2012	17/20 patients	IIV Fosfluconazole, micafungin, liposomal amphotericin B, and voriconazole, Oral itraconazole, fluconazole, voriconazole	ID pharmacist intervention regarding culture reports and antimicrobial use based on guidelines	30-day mortality	30-day mortality rates pre-/post-AFS group was 29.4% vs. 60% (*p* = 0.099) NS.
Amanati et al. (14)	Cross-sectional	Tertiary Teaching Hospital, Sheraz, Amir Medical Oncology Center (Iran)	Children aged ≤18 years with hematologic malignancy or solid tumor	(2011–2012) (2017-2018)	136 patients	Amphotericin B, caspofungin, voriconazole, itraconazole, fluconazole	AF susceptibility testing, adherence to guidelines, application of non-culture-based methods GM, PCR	Impact of AFS on AF susceptibility patterns of colonized *Candida* sp.	The most prevalent strain was *Candida albicans.* Resistance to azoles was significantly reduced from 52.5 to 1.5% (*p* < 0.001), caspofungin resistance reduced from 10.1 to 0.9%, fluconazole resistance reduced from 32.9 to 0.0% (*p* < 0.001).
Kara et al. (27)	Prospective quasi-experimental	1,040-bedded Tertiary Care University Hospital (Turkey)	Adult patients ≥18 years receiving systemic antifungals	2019–2020	84/101/192 patients	Fluconazole, voriconazole, posaconazole, caspofungin, micafungin, Anidulafungin, Liposomal Amphotericin B and combinations	Feedback/education to physicians, evaluation of appropriateness of AF, and adherence to guidelines	Adequacy of AF therapy, pDDI, and 30-day mortality	Appropriateness of AF use increased significantly (*p* < 0.001). The acceptance rate of recommendations was 96% (151/157). pDDI decreases significantly (*p* = 0.035) and 30-day mortality reduced (*p* = 0.05).
Markogiannakis et al. (38)	Prospective quasi-experimental interrupted time series analysis	535-bed tertiary care teaching hospital, General Hospital of Athens Laiko (Greece)	Adult patients ≥18 years with hematological/oncological malignancy	2015–2017	147 + 138 (285) patients	Azoles, polyenes, and echinocandins antifungals	Educational intervention, prospective audit, and feedback	Quality of prescriptions, in-hospital-mortality, and in-hospital length-of-stay	A significant increase in the appropriateness of prescriptions 47% → 76.2% (*p* = 0.01) All-cause in-hospital mortality reduced from 39.5 → 37.1, and LOS reduced from 5.19 → 4.96 (NS)
Gamarra et al. (22)	Prospective quasi-experimental	250 bedded a university-affiliated tertiary care hospital, Hospital Universitario Clementino Fraga Filho (Brazil)	Patients admitted to the hematology ward, infectious diseases ward, internal medicine and ICU	2016–2018	270 Patients	Fluconazole, amphotericin B (deoxycholate, lipid complex and liposomal), voriconazole, posaconazole, and echinocandins	Educational intervention using charts followed by a retrospective audit	Adequacy of AF use	Significant reduction of inappropriate prescriptions (80.2%) in the first audit vs. 64.6% in the second audit (*p* = 0.001).
Bio et al. (18)	Retrospective cohort	Children hospital (America)	In-patient children <18 years	2020–2022	1,803 prescriptions	Fluconazole, posaconazole, isavuconazole, and echinocandins	Prospective audit and feedback	Recommendation rate and acceptance	Among 379 recommendations, 298 were accepted (78.62%). Among all, discontinuation of AF was the most common.
Keck et al. (29)	Prospective quasi-experimental	University of Mississippi Medical Center (Mississippi)	In-patients ≥18 years who had received at least one treatment dose of micafungin	2020–2022	282 patients	Micafungin	ID consult via prospective audits and feedback.	Days of micafungin therapy, LOS, in-patient mortality, time to de-escalation, and discontinuation after 72-h time out	Duration of micafungin treatment decreased (*p* = 0.005), whereas, LOS (*p* = 0.137), in-patient mortality (*p* = 0.637), and micafungin discontinuation (*p* = 0.788), or de-escalation (*p* = 0.530) remains NS.

*p<0.05 indicates significant results. DDD, Defined daily dose; DOT, Days of therapy; NS, Non-significant; AFS, Antifungal stewardship; LOS, Length of hospital stay; ID, Infectious disease; pDDI, potential drug–drug interaction; IV, Intravenous; GM, Galactomannan; PCR, Polymerase chain reaction; AF, Antifungal.*

All studies were single-centered except one that was conducted in five different hospitals. Among all included studies evaluating antifungal consumption, four studies evaluated DDD/1,000 patient days, and three studies measured DOT/1,000 patient days. Two studies measured DDD/100 patient or bed days (3, 13–18). Five studies were prospective quasi-experimental in design and conducted time series analysis, while two studies were observational, and 1 was prospective cohort. All studies have variable duration, with a least 18 months and a maximum duration of 10 years. Pharmacist interventions include educational interventions, prospective audits and feedback, and focusing adherence to guidelines.

Among studies evaluating clinical outcomes, seven studies were prospective, quasi-experimental in design, five studies were retrospective observational, five studies were prospective cohort, one was retrospective cohort, and one was cross-sectional in design. All studies were single-centered except one that evaluated clinical outcomes of AFS in five hospitals. The majority of studies were carried out on adult patients >18 years of age, and the maximum duration of the study was 7 years, and the minimum duration was 2 years (1, 2, 4–7, 9, 19–30), as shown in Table 1.

### 3.3 Interventions

Pharmacist interventions vary among the included studies. In five of the eight studies evaluating the impact of the consumption of antifungals, pharmacist interventions were based on educational activities that included academic detailing inculcating prospective audits and feedback (3, 13, 15, 17, 18). Other interventions include recommendations about diagnostic tests, TDM-based dosing and stopping treatment in patients without confirmed diagnosis (14), appropriate selection and modification of therapy (16), and optimizing antifungal therapy based on culture reports and guideline recommendations (18).

Nineteen studies that evaluated the impact on clinical outcomes, namely, pharmacist interventions included the development of candidemia care bundle based on Infectious Diseases Society of America (IDSA) guidelines (19, 22), implementing antifungal (AF) susceptibility testing, culture alerts, initiation of echinocandins, removal of central lines (20), academic detailing (audit and feedback) regarding appropriate empiric regimen, tailoring and reassessment (2, 4, 29, 30), therapeutic drug monitoring (4, 5, 23, 25), and infectious disease referrals (1, 4) along with *β*-d-glucan (βDG) measurement (7, 9), recommendation according to European Society of Clinical Microbiology and Infectious Diseases (ESMID) guidelines for candidemia management (21), medical training for the physicians, pocket card summarizing recommendations for antifungal use and on-ward pharmaceutical counselling (6), stopping empirical antifungal after 72 h, automated alert and ID consult for empirical antifungals and switching from intravenous (IV) to oral fluconazole (1), ID pharmacist intervention regarding culture reports (26, 27), adherence to guidelines, application of non-culture based methods Galactomannan, polymerase chain reaction (PCR) (27), feedback/education to physician (28) are shown in Table 1.

### 3.4 Antifungal consumption

Eight studies evaluated the impact of pharmacist interventions on antifungal consumption. Consumption metrics used were variable, and few studies involved more than one metric. Three studies measured DDD/1,000 patient days (3, 16, 18), One study measured DDD/100 bed days (14), one measured DDD/100 patients (15), three measured DOT/1,000 patient days (3, 16, 18), and one measured DDD/1,000 bed days (17). Due to the variability in measuring units, a quantitative estimation was not possible; however, DDD/1,000 patient days significantly declined (62%; *p* = 0.009; *p <* 0.001); a significant reduction in DDD/100 bed days (*p* < 0.017); a significant decrease in DDD/100 patients (*p* < 0.01); DOT/1,000 patient days remained insignificant in one study and a significant decrease in one study (*p* < 0.001) was evident; and one study showed an increase in DOT/1000 patients for anidulafungin and caspofungin while declining in fluconazole and L-amphotericin B as a result of pharmacist interventions (Table 1).

### 3.5 Clinical outcomes

Clinical outcomes that were evaluated post-AFS implementation included mortality, the length of hospital stay, the occurrence of adverse events, and the quality of antifungal use (Table 1).

### 3.6 Mortality

The impact of stewardship interventions was evaluated on 30-day, 60-day, and 14-day mortalities and on in-patient mortality in the included studies. Five studies reported the data of 30-day mortality, in which four showed non-significant results pre-/postintervention (19, 21, 22, 26) and one study showed a significant decline in 30-day mortality post-AFS (*p* = 0.05) (28). Furthermore, 60-day, 90-day, and 14-day mortalities pre-/post-AFS also remained non-significant (7, 21, 24). In-hospital/in-patient mortality was decreased significantly in one study (*p* = 0.054) (22) and non-significantly in two studies (9, 29).

### 3.7 Length of hospital stay

Five studies measured the length of hospital stay as an outcome of antifungal stewardship, and in all included studies post-AFS, there is a non-significant change in the length of hospital stay (4, 7, 9, 20, 29).

### 3.8 Adverse events

Four studies evaluated the role of pharmacist interventions on the occurrence of adverse drug events. One study showed that pharmacist interventions resulted in a significant decline in adverse events (*p =* 0.004) (7), and one study showed a significant increase in preventable adverse drug events post-AFS 58 vs. 230 (23). The results of another study evaluating TDM-based dosing identified that the drug discontinuation due to hepatotoxicity and visual symptoms was 62.5 and 26.3%, respectively (5), and one study identified that potential drug–drug interaction (pDDI) decreased significantly as a result of pharmacist interventions (*p* = 0.035) (28).

### 3.9 Quality of antifungal use

Quality of antifungal use encompasses appropriate drug selection, referrals for ID consultation, implementing culture tests, and adherence to guidelines. Fifteen studies evaluated different parameters of the quality of antifungal use post-AFS. Seven studies (1, 6, 19, 25, 28–30) evaluated appropriate drug selection, and all studies showed significant improvement in antifungal selection (*p* < 0.001, *p* < 0.005, *p* < 0.0001, *p* = 0.0001, *p* < 0.001, *p* = 0.01, and *p* = 0.001) respectively. The result of one study showed that repeat blood cultures were improved significantly (*p* = 0.012 and *p* = 0.04) (19, 21). One study reported a significant increase in ID consultation (*p* < 0.001) (20), and another study reported an increase from 36.4 to 86.5% (21). One study reported data on the resistance rate to azoles post-AFS, which was significantly declined (*p* < 0.001) (27), and the incidence of hospital-acquired candidemia was also decreased (*p* = 0.009). Three studies evaluated adherence to guidelines. One study showed significant adherence to IDSA guidelines (19); another study reported that achievement and adherence to the candidemia care bundle were significant postintervention (*p* = 0.006) (22); and one study showed significant adherence to clinical guidelines for antifungal prophylaxis (*p* = 0.0344) (25) as shown in Table 1.

### 3.10 Antifungal expenditure

Seven studies reported the impact of pharmacist intervention on antifungal expenditure or cost savings. Among all, the maximum reduction in antifungal expenditure was 73% (13), and the least reduction in cost was 13.5% (16). Two studies reported a significant decline in antifungal cost (*p* = 0.03 and *p* = 0.002), as shown in Table 1. An overall summary of study characteristics is shown in Table 2.

**Table 2:** Summary of study characteristics.

Study characteristics	Number of studies	Study reference
Age group		
Adult	22	(1–7, 9, 13–15, 17–20, 23–26, 28–30)
Pediatric	2	(2, 27)
Both	3	(16, 21, 22)
Patient type		
Inpatient	26	(1–7, 9, 13–24, 26–30)
Outpatient	1	(25)
Country		
High-income country	23	(1, 2, 4–7, 9, 13–26, 29)
Low-income country	4	(3, 27, 28, 30)
Study design		
Prospective, quasi-experimental	12	(3, 9, 13, 14, 17–20, 24, 28–30)
Retrospective observational	7	(4–6, 15, 16, 22, 23)
Prospective cohort	6	(1, 7, 18, 21, 25, 26)
Retrospective cohort	1	(2)
Cross-sectional	1	(27)
Type of intervention		
Educational intervention, audit, and feedback	15	(2–4, 6, 7, 9, 13, 15, 17–19, 24, 28–30)
Pharmacist recommendation for therapeutic drug monitoring, fungal markers, Antifungal susceptibility testing, and blood culture	14	(1, 5, 9, 14, 16–18, 20–23, 25–27)
Outcomes		
Antifungal consumption	8	(3, 13–18)
Antifungal expenditure	7	(13–16, 18, 23)
Mortality	8	(4, 7, 9, 19, 21, 22, 24, 26)
Length of hospital stay	4	(4, 9, 20, 23)
Decline in clinical failure	2	(1, 7)
Referral for ID consults	2	(20, 21)
Quality of antifungal use	8	(2, 6, 9, 21, 25, 28–30)
Reduction in adverse drug events	4	(5, 7, 23, 28)
Reduction in resistance rate	1	(27)

## 4 Discussion

This review aims to evaluate the role of AFS pharmacists in optimizing antifungal therapy and its impact on antifungal usage, consumption, and clinical outcomes globally. The majority of healthcare systems lack proper diagnostic techniques, and suboptimal levels of antifungal drugs with non-linear kinetics result in treatment failure (21). Literature has shown that pharmacist-driven AFS can enhance the appropriateness of antifungal treatments by improving the selection of drugs, dosages, and therapy durations, while also preventing potential drug interactions (27).

The stewardship approach was variable among all included studies, but the most common pharmacist intervention was academic detailing with audit and feedback followed by dose adjustments based on therapeutic drug monitoring. Prospective audits and feedback in hospital settings have been demonstrated to enhance the quality of prescription practices and are advocated as a vital component of antifungal stewardship (18). Medical training for physicians and on-ward pharmaceutical counseling regarding antifungal utilization can be crucial in securing a sustained impact of an interdisciplinary AFSP (9). Previous studies have shown that prescribers could not differentiate between fungal colonization and underlying disease, as well as the appropriate use of prophylactic vs. empirical antifungal medication (31). Therapeutic drug monitoring (TDM) plays a crucial role in optimizing antifungal therapy, with routine recommendations for voriconazole monitoring outlined in the guidelines of the British Society for Medical Mycology and the IDSA (15). Voriconazole, being the first-line drug for invasive aspergillosis, exhibits non-linear pharmacokinetics, where both high and low serum concentrations are associated with increased risks of hepatotoxicity and therapeutic failure, respectively (25). Study results showed that with TDM-based dose adjustment, the treatment completion rate was increased (8.8%) (23). Another study reported that the major reason for non-adherence was a lack of TDM (32). Implementing TDM practices within healthcare institutions can enhance the effectiveness and safety of antifungal therapy by ensuring optimal drug exposure for each patient. Antifungal stewardship teams involving pharmacists in hospitals led to a notable decrease in the consumption and acquisition costs of antifungals (30). Mycoses Study Group Education and Research Consortium recommended that Stewardship team core members should possess a deep understanding of fungal epidemiology and susceptibility patterns, laboratory diagnosis of IFD, the spectrum, and pharmacokinetics of antifungal drugs, strategies for optimizing dosing and duration, fungal surveillance, and the ability to anticipate, interpret, and manage drug–drug interactions and antifungal toxicities. Furthermore, proficiency in interpreting therapeutic drug monitoring is essential. Ideally, the team should include infectious diseases (ID) physician(s) and ID-trained pharmacist(s) whenever feasible (26).

The impact of stewardship intervention was evaluated on the consumption of antifungals. All included studies utilize variable matrices DDD/100 patient days, DOT/1,000 patient days, or DDD/1,000 bed days. However, overall, antifungal consumption decreases (3, 13–18) and results in a cost reduction of up to 74.7 (23), with another reporting 73% (13). In a systematic review conducted in 2017 on AFS interventions and performance measures, it was noted that antifungal consumption exhibited a decrease ranging from 11.8 to 71% and a reduction in expenditure of 50% (17). AFS programs aim to strike a balance between effective treatment and prudent use of antifungal agents, decreasing overall antifungal consumption. Pharmacist interventions are crucial in reducing antifungal consumption by optimizing therapy, implementing evidence-based guidelines, utilizing therapeutic drug monitoring (TDM), providing education and training, and transitioning patients from intravenous to oral antifungals when appropriate. These strategies help ensure the judicious use of antifungal agents, ultimately reducing both consumption and associated healthcare costs (9). Antifungal drugs are expensive; their judicious use promotes better resource allocation, reduces healthcare costs, and improves patient outcomes. Optimization of antifungal therapy by ensuring appropriate selection, dosing, and treatment duration, not only curbs unnecessary spending but also lowers the risk of antifungal resistance, ultimately enhancing the quality of care for patients with fungal infections (13).

Another parameter evaluated in this review was clinical outcomes including mortality, the length of hospital stay, the occurrence of adverse events, and the quality of antifungal prescribing or use. Major interventions of pharmacists include academic detailing, tailoring drug therapy based on culture reports, and conducting therapeutic drug monitoring. In-hospital/patient mortality decreased significantly in one study (22), and one study showed a significant decline in 30-day mortality post-AFS (28), while all other included studies reported a non-significant decline in mortality and length of hospital stay. Although it was non-significant, overall, AFS decreases mortality and length of hospital stay. However, the exact estimate is not possible as the underlying disease may be associated with early mortality. More studies are required to further strengthen this evidence. Moreover, heterogeneity of patient population, diverse clinical presentation and variable treatment response contribute to the difficulty in providing precise estimates for mortality and LOS in immunocompromised patients with systemic fungal infections. Four studies identifying the impact of adverse events reported a significant decline in adverse event occurrence and potential drug–drug interactions (5, 7, 23, 28). AFS programs involve regular monitoring of patients on antifungal therapy. This surveillance helps promptly identify and address adverse events, contributing to improved patient safety (27).

Quality of antifungal use evaluated post-AFS in 15 studies includes appropriate prescribing, culture-based drug tailoring, and adherence to guidelines for fungal infection management. All included studies reported significant improvement in antifungal prescribing, culture evaluation for targeting therapy, and adherence to guidelines. Diagnostic precision and optimized dosing overall improve the quality of antifungal use (19).

## 5 Conclusion

Pharmacist-led AFS has the potential to enhance the effectiveness of antifungal treatments by improving their overall quality and reduction in antifungal consumption, adverse events, and antifungal expenditure. Moreover, novel fungal diagnostic techniques, TDM, and antifungal susceptibility testing must be integrated with AFS in hospitals to rationalize antifungal use and decrease the emerging threat of antifungal resistance.

### 5.1 Strength and limitation

Systematic reviews focusing on pharmacist-driven antifungal stewardship interventions after 2018 are scarce. Thus, we collected literature from PubMed and Google Scholar databases to present and evaluate the published evidence in the last 5 years (2018–2023). Due to limited institutional access to other resources such as Embase and Scopus, data collection was restricted only to two databases. The major limitation is variation in interventions and outcome metrics; hence, an integrative review approach was utilized. Variations in healthcare settings may limit the generalizability of the findings. All primary studies used descriptive statistics for their evaluations, presenting findings as frequencies and percentages without employing an effect measure estimate; therefore, results were presented descriptively.

### 5.2 Future perspective

Future research should investigate the influence of pharmacist-led stewardship in outpatient clinics and community settings. In addition, further studies are also warranted on the pediatric population using the DOT methodology to provide a more comprehensive and standardized assessment of pharmacist interventions on consumption as well as clinical outcomes. Detailed subgroup analyses based on specific healthcare settings and intervention types are also required to elucidate how differences in healthcare settings and types of interventions impact clinical outcomes.

## Data Availability

The original contributions presented in the study are included in the article/supplementary material, further inquiries can be directed to the corresponding author.
